# Multimodal magnetic resonance imaging after experimental moderate and severe traumatic brain injury: A longitudinal correlative assessment of structural and cerebral blood flow changes

**DOI:** 10.1371/journal.pone.0289786

**Published:** 2023-08-07

**Authors:** Juliana Sanchez-Molano, Meghan O. Blaya, Kyle R. Padgett, William J. Moreno, Weizhao Zhao, W. Dalton Dietrich, Helen M. Bramlett

**Affiliations:** 1 Department of Neurological Surgery, The Miami Project to Cure Paralysis, University of Miami Miller School of Medicine, Miami, Florida, United States of America; 2 Department of Radiation Oncology, University of Miami Miller School of Medicine, Miami, Florida, United States of America; 3 Department of Biomedical Engineering, University of Miami Miller School of Medicine, Miami, Florida, United States of America; 4 Bruce W. Carter Department of Veterans Affairs Medical Center, Miami, Florida, United States of America; University of Florida, UNITED STATES

## Abstract

Traumatic brain injury (TBI) is a worldwide problem that results in death or disability for millions of people every year. Progressive neurological complications and long-term impairment can significantly disrupt quality of life. We demonstrated the feasibility of multiple magnetic resonance imaging (MRI) modalities to investigate and predict aberrant changes and progressive atrophy of gray and white matter tissue at several acute and chronic time points after moderate and severe parasagittal fluid percussion TBI. T2-weighted imaging, diffusion tensor imaging (DTI), and perfusion weighted imaging (PWI) were performed. Adult Sprague-Dawley rats were imaged sequentially on days 3, 14, and 1, 4, 6, 8, and 12 months following surgery. TBI caused dynamic white and gray matter alterations with significant differences in DTI values and injury-induced alterations in cerebral blood flow (CBF) as measured by PWI. Regional abnormalities after TBI were observed in T2-weighted images that showed hyperintense cortical lesions and significant cerebral atrophy in these hyperintense areas 1 year after TBI. Temporal DTI values indicated significant injury-induced changes in anisotropy in major white matter tracts, the corpus callosum and external capsule, and in gray matter, the hippocampus and cortex, at both early and chronic time points. These alterations were primarily injury-severity dependent with severe TBI exhibiting a greater degree of change relative to uninjured controls. PWI evaluating CBF revealed sustained global reductions in the cortex and in the hippocampus at most time points in an injury-independent manner. We next sought to investigate prognostic correlations across MRI metrics, timepoints, and cerebral pathology, and found that diffusion abnormalities and reductions in CBF significantly correlated with specific vulnerable structures at multiple time points, as well as with the degree of cerebral atrophy observed 1 year after TBI. This study further supports using DTI and PWI as a means of prognostic imaging for progressive structural changes after TBI and emphasizes the progressive nature of TBI damage.

## Introduction

Traumatic brain injury (TBI) is a critical global health problem that results in death or disability for millions of people every year [1]. TBI is a causal factor in 34 percent of all injury-related deaths in the United States [2]. In addition, The Center for Disease Control and Prevention [3] reported that 2.8 million individuals sustain a TBI each year. Falls, motor vehicle collisions, sports-related injuries, abuse/assault, and blast-induced high-pressure waves are the primary causes of TBI [2].

TBI can result in an extensive range of deleterious consequences, from cognitive deficits and disabling neuropsychiatric symptoms to coma and death [4–6]. Unfortunately, the full extent of symptomatology is often not fully realized until several years after the injury due to the progressively degenerative nature of TBI and evolving histopathological changes [7]. Additionally, TBI has been reported to be a risk factor for the development of Alzheimer’s disease (AD) and other neurodegenerative disorders [4, 7–10].

Previous clinical and preclinical studies have reported progressive atrophy of specific gray and white matter structures after various types of TBI [8, 11, 12]. These progressive changes are associated with chronic deficits in a number of neurological disorders [13–18]. In clinical studies, anatomical injuries resulting from TBI include contusions in highly susceptible regions, such as the frontal and temporal lobes [19]. In addition, opposing pressure gradients generated by the primary mechanical injury can alter vulnerable tissue microarchitecture. Stretching, compression, and rotation of susceptible tissue often results in multi-focal white matter abnormalities and diffuse axonal injury (DAI)/traumatic axonal injury (TAI), leading to progressive alterations in axonal transport and function [20]. These phenomena occur hours to days after injury, possibly creating multiple therapeutic windows for treatment [11, 21–23]. Recent studies have shown that DAI is correlated with long-term consequences associated with neurodegenerative processes that can persist for years after the injury and may contribute to deleterious neurological events such as Alzheimer’s disease-like pathological changes [10, 24].

Neuroimaging is essential in diagnosing, classifying, and for the management of TBI patients. Focal contusions and lesions can be visualized using computed tomography (CT) scan and conventional magnetic resonance imaging (MRI), however the visualization of these acute injuries is often unable to predict clinical outcomes and is not always sufficiently sensitive to detect microscopic secondary changes after TBI [25–29]. One explanation for this gap in imaging and functional outcomes is that conventional imaging techniques miss the presence of DAI, and the disruption of white matter integrity and subsequent alterations in more remote subcortical regions are not fully appreciated with single imaging approaches, which contributes to the degree of deficit severity and correlates to overall functional outcomes. Observation of white matter hemorrhages in neuroimaging is interpreted as coincident with DAI, however 80% of axonal injuries are non-hemorrhagic [30]. Skandsen et al. reported DAI was observed in 72% of patients presenting with moderate to severe head injury [31].

Diffusion tensor imaging (DTI) is an advanced non-invasive MRI technique that provides critical information regarding white matter tract integrity by measuring water diffusion and changes in anisotropy [26, 32, 33]. Parametric maps, including fractional anisotropy (FA), apparent diffusion coefficient (ADC), and radial diffusivity (RD), allow for the determination of the directional flow and permissibility of water molecule diffusion through tissue and hence the subsequent integrity or compromise of white matter [34].

ADC reflects the diffusion speed of water molecules and can be utilized to determine the degree of obstruction of water molecule diffusion in turn providing information regarding histological architecture [35]. In DTI, changes in FA can be reflective of alterations of several elements, such as axonal membrane integrity, myelination, axon diameter, and axon attenuation [36]. Alterations in FA are significantly correlated with cognitive deficits in moderate and severe TBI patients, and have been recognized as one of the most sensitive markers of TBI-induced cognitive and motor impairment [37]. RD is a scalar measurement of diffusion that is perpendicular to the length of an axon, and it, along with FA, are considered proxies for fiber organization and integrity of myelination. RD values are also highly sensitive to DAI and TAI [38].

Hemodynamics and cerebrovascular changes are observed after TBI as preclinical studies have reported impaired cerebral vascular autoregulation, microvascular injury, and increased blood-brain barrier permeability, resulting in changes of cerebral blood flow (CBF), ischemia, and infarction [39–43]. Kochanek and colleagues reported a significant reduction in cortical and hippocampal CBF 1 year after controlled cortical impact injury, which was concomitant with tissue loss in these structures [44]. Microvascular abnormalities at various posttraumatic time periods after TBI are associated with DAI and in subsequent cognitive and functional impairment [45, 46]. Therefore, another valuable, non-invasive imaging modality is perfusion-weighted imaging (PWI), which provides information on the hemodynamic state of the brain and allows for the detection of alterations in perfusion of the injured area and penumbral regions of the brain.

In the present study, we sought to investigate the ongoing and progressive nature of structural and hemodynamic consequences after moderate and severe TBI using an established model that produces a pressure-induced gliding contusion at the gray/white matter interface resulting in both focal and diffuse gray and white matter injury [47]. Through the longitudinal assessment of DTI and PWI alterations we investigated how those changes are reflected in chronic atrophic tissue at 12 months post injury. We propose that using both DTI and PWI modalities at several time points will provide an additional level of analysis and better illuminate the degree of neurological dysfunction after trauma and thus identify both early and later occurring cellular and physiological therapeutic targets for TBI.

## Materials and methods

### Animals

Adult 3- to 4-month-old male Sprague Dawley rats (*Rattus norvegicus*; n = 26) were randomly assigned to 1 of 3 groups: uninjured sham (n = 10), moderate TBI (n = 8), or severe TBI (n = 8). Blocking randomization and standardization strategies were used to enhance scientific rigor and to control for variables that could potentially bias the results and ensure balanced numbers in each treatment group [48]. Females were not utilized in the present study as hormonal fluctuations associated with the female reproductive cycle may introduce biological variability. To standardize TBI models, monitoring and controlling for physiological and metabolic variables is important to reduce variability. In accordance with this, to determine the minimum number of animals needed for these studies and obtain meaningful data, a sample size analysis using SigmaPlot software 14.5 was performed for each outcome measure. Power was set to 0.80, minimum detectable difference in means, expected standard deviation of residuals, number of groups and alpha at 0.05 were calculated for each outcome to determine sample size. A sample size of n = 8–10 per group was obtained. All experimental procedures were compliant with the National Institutes of Health *Guide for Care and Use of Laboratory Animals* and approved by the University of Miami Institutional Animal Care and Use Committee, and in compliance with Animal Research Reporting In vivo Experiments (ARRIVE) guidelines. Animals were housed in a temperature-controlled room (22° C) and exposed to a 12 h light/dark cycle. They were acclimated for at least 7 days prior to surgery.

### Fluid-percussion injury

On Day 1 of surgery, animals were anesthetized with 3.0% halothane and a mixture of 70% nitrous oxide (N_2_O) and 30% oxygen (O_2_). Once an adequate level of anesthesia was reached, the animals were placed in a stereotaxic frame. A 4.8 mm craniotomy was made overlying the right parietal cortex (3.8 mm posterior to bregma and 2.5 mm lateral to the midline), and a plastic injury tube (3.5 mm inner diameter) was adhered to the skull over the intact dura. The scalp was then closed, and animals were allowed to recover before being returned to their home cage. At this point, food was removed, and animals were fasted overnight to maintain consistent glucose levels, which can affect TBI outcomes [49, 50].

Twenty-four hours later, animals were reanesthetized with 3.0% halothane in a mixture of 70% N_2_O and 30% O_2_. The right femoral artery was cannulated (PE-50 tubing), and blood samples were collected at regular intervals to monitor blood gases (pO_2_ and pCO_2_), blood pH, glucose, and mean arterial blood pressure (MABP). Rats were intubated, immobilized with pancuronium bromide (1.0mg/kg IA), mechanically ventilated, and maintained on 0.5–1.0% halothane in a mixture of 70% N_2_O, and 30% O_2_. For TBI groups, an FPI device was used to induce moderate (1.8–2.2 atmosphere) or severe (2.3–2.5 atmosphere) parasagittal FPI [40, 51]. Physiological variables were maintained in normal ranges 15 min prior to TBI and for up to 30 min post-injury (*p*O_2_, 105–140 mmHg; *p*CO_2_, 35–45 mmHg; pH, 7.35–7.45), and brain and body temperatures were maintained at 37°C using a feedback heat lamp system. Pain management measures were taken to alleviate discomfort. Buprenorphine was given once after surgery (0.01 mg/kg, subcutaneously). Sham animals underwent identical procedures minus the physical fluid percussion insult. Animals were allowed to survive for 1 year and were then sacrificed for histological analysis.

### Physiology

All physiological variables, including pH, pO_2_, pCO_2_, and MABP were maintained within normal ranges before and after FPI. Animals showed normal activity within 24 h after recovery from anesthesia, which was determined by observation of grooming behavior, posture, and locomotion. There was no significant weight loss in any experimental group. No animals were excluded after surgery.

### Magnetic resonance imaging

Magnetic resonance measurements were performed on each animal during critical early time points (Days 3 and 14) and at several chronic time points (1, 4, 6, 8, and 12 months) post-surgery utilizing a 4.7-Tesla (200-MHz) 30-cm bore magnet interfaced to a Bruker Advance console. The investigators were blinded to the condition of the animal. During the MRI sessions, animals were anesthetized with 1.0% halothane in N_2_O/O_2_ (70%/30%) and placed in an MRI-compatible cradle in the prone position utilizing a custom-built plexiglass holder with the head and nose securely fixed to minimize motion artifacts. Warm water (37 ± 0.5°C) was circulated through the animal cradle to control the temperature of the animals. Volumetric measurements of the brain were performed by utilizing a high-resolution T_2_ rapid acquisition relaxation-enhanced (RARE) multi-slice acquisition. Twenty-five slices were acquired to include all structures used in the histopathological analysis. The imaging parameters were as follows: a RARE factor of 8, TR4500 ms, effective TE 67.5 ms, field of view 3.00 cm, image matrix size of 184X184 (163 μm in-plane resolution) and slice thickness of 500 μm. Additionally, DTI datasets were acquired utilizing 8 independent diffusion directions, employing a b-value of 0 and 1000s/mm^2^, a TR of 1500 ms, a TE of 29 ms, a matrix size of 128x128 (234 μm in-plane resolution) and a slice thickness of 1 mm. Lastly, continuous arterial spin labeling (cASL) EPI perfusion datasets were collected to calculate CBF maps of the brain. A 4-shot segmented EPI scan was used for the cASL experiments utilizing a TE of 50 ms, a TR of 1800 ms, a field of view of 3.0 cm, image matrix size of 128X128 (234 μm in-plane resolution) and slice thickness of 1.5 mm. The labeling settings employed for the cASL studies were a 1500 ms labeling period utilizing a 200 ms post-labeling delay and a 10mT/m labeling gradient. The total time for imaging was 1 h 30 min per session.

### Volumetric analysis (T2 datasets)

Regions of interest (ROIs) were manually defined on the T2-weighted datasets on the Bruker Advance console by a blinded investigator [52]. A program was developed to read in polygan convex coordinates of the ROIs to export the reconstructed ROIs from the Bruker console to different computer platforms. Based on different scanned slice thickness (T2: 0.5 mm, DTI: 1 mm, PWI: 1.5 mm), we selected the T2 sections corresponding to the same anatomical sections of the DTI/PWI images and superimposed reconstructed ROIs on top of the DTI/PWI images to acquire ROI data in terms of mean and standard deviation.

### Diffusion tensor analysis

DTI datasets were analyzed by a blinded investigator using DTI-Studio^™^ created by Susumu Mori et al. at Johns Hopkins University [53]. This software was employed to create ADC maps, FA maps, RD [34] maps and principle Eigen value maps (EVA0) maps. ADC and RD metrics are presented in mm^2^/s. FA is expressed in arbitrary units (a.u.). ROIs were first defined on the T2 weighted image obtained during the same imaging session and at the same neuroanatomical level as the diffusion-weighted images (DWI), and then transferred onto the DTI parameter maps.

### Perfusion weighted imaging

PWI datasets were generated by the Bruker scanner. Based on the published method [54], using water as a freely diffusible tracer, we present the CBF images measured in unit of ml/g/minute [46].

### Quantitative histopathology

At 1-year post TBI or sham procedures, animals were reanesthetized with 3% halothane in 30% O_2_ and 70% N_2_O, and transcardially perfused with saline (80 ml for 2 min) followed by 4% paraformaldehyde (4°C, 350 ml at a pressure of 100–120 mm Hg for 28 min) by a surgeon blind to the condition of the animal. After decapitation, the brains were immediately removed and placed in 4% paraformaldehyde at 4°C for 48 h. Brains were then blocked and embedded in paraffin for tissue sectioning. Coronal tissue sections were acquired at 10 μm thickness on a microtome (Leica HM 2125) at 150 μm intervals. One series of sections were stained with Luxol Fast Blue and hematoxylin-eosin (H&E) for volumetric assessment. Brain volume was measured by contouring the ipsilateral and contralateral cortex, corpus callosum (CC), external capsule (EC), and hippocampus at 7 specific bregma levels (-0.8, -1.8, -3.3, -4.3, -5.8, -6.8-, and -7.3-mm posterior to bregma) [55]. Volumes were determined by tracing the boundaries of each structure at a power of 10x from a minimum of two coronal sections at each of the bregma levels, using a camera lucida microscope attachment. To determine the differences in tissue shrinkage, percent atrophy was determined by calculating the difference between ipsilateral and contralateral volume and normalizing to the contralateral volume. Representative images were acquired at 20x using an Olympus BX51 microscope (Olympus America), and the volume estimation was carried out using Neurolucida software (version 7.50.1, Micro Brightfield Inc., Willinston, ME) by a blinded investigator.

### Statistical analysis

All statistical analysis procedures were blindly conducted on GraphPad Prism (v 9.5.0). Data are expressed as mean ± standard error of the mean (SEM). Intergroup differences among bilateral structural volumes, DTI measurements, and PWI values were evaluated using one- or two-way repeated measures analysis of variance (ANOVA). In the case of missing data points, mixed-effects model analysis is conducted. The Shapiro-Wilk test (alpha = 0.05) was applied to determine if data were normally distributed and Bartlett’s test to determine if standard deviations were significantly different. Post-hoc multiple comparison analysis was performed using Tukey corrections to control for the probability of making at least one Type 1 error across all pairwise comparisons. Significance level was p < 0.05. Exact p-values, Mean differences, SE of differences, q statistics, degrees of freedom (DF), 95% CI of ipsilateral differences are reported in the text and in entirety in S1 Table. Correlational analyses were carried out comparing all ADC, RD, and FA metrics to PWI CBF and histopathological indices within each structure at each imaging time point across groups. Correlations between parameters that were significant were determined using simple linear regression analysis and r Pearson values reported. Only significant correlations were reported. Full regression analysis results are reported in S2 Table.

## Results

**Fig 1:**
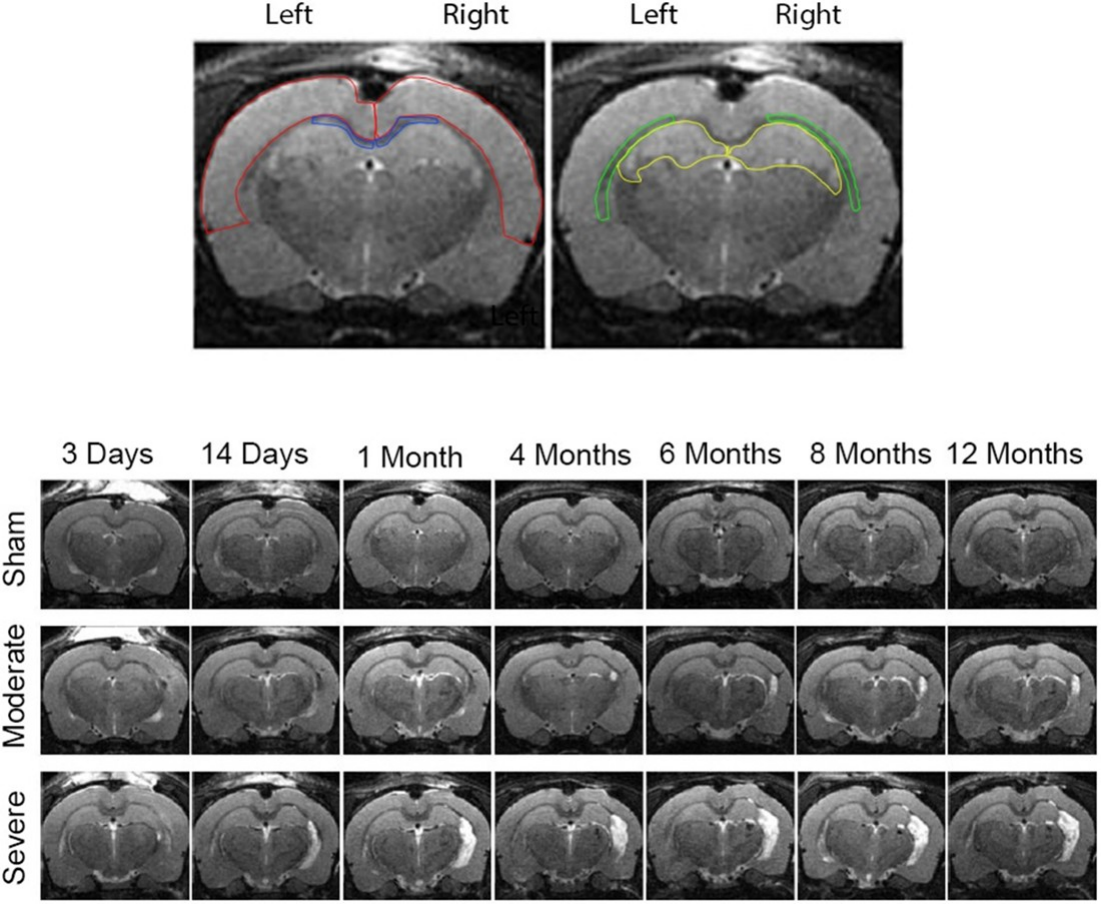
Magnetic resonance imaging (MRI) scans depicting ROIs and longitudinal scans of all groups across time points. **(Top)** Regions of interest (ROIs) were defined using the T2-weighted datasets of the ipsilateral (right) and contralateral (left) side of the brain for the following areas: cerebral cortex (outlined in red), hippocampus (yellow), external capsule (green) and corpus callosum (blue). (**Bottom)** T2-weighted images from a representative sham, moderate TBI, and severe TBI animal imaged longitudinally at 7 different time points. Progressive cerebrostructural changes were observed in several different areas across time points corresponding to the temporal evolution of the injury as indicated by hyperintense signal (white region) in the ipsilateral side. Moderately and severely injured animals developed enlarged lateral ventricles in the injured hemisphere where the cortical lesion was present relative to the contralateral non-injured hemisphere. Chronic images of severe TBI animals also revealed thinning of cortical tissue and shrinkage of ipsilateral hippocampus.

Vulnerable regions are affected in TBI, resulting in hallmark sequelae across patients. We investigated key ROIs susceptible to functional and structural damage after brain injury (Fig 1, **top**). After undergoing moderate or severe fluid-percussion TBI or sham surgery, animals were imaged longitudinally 7 consecutive times using described multiple MRI modalities. Across time points, T2-weight images illustrated progressive anatomical pathology in both injury groups within the injured ipsilateral (right-hand side) hemisphere (Fig 1, **bottom**).

### Diffusion tensor imaging

**Fig 2:**
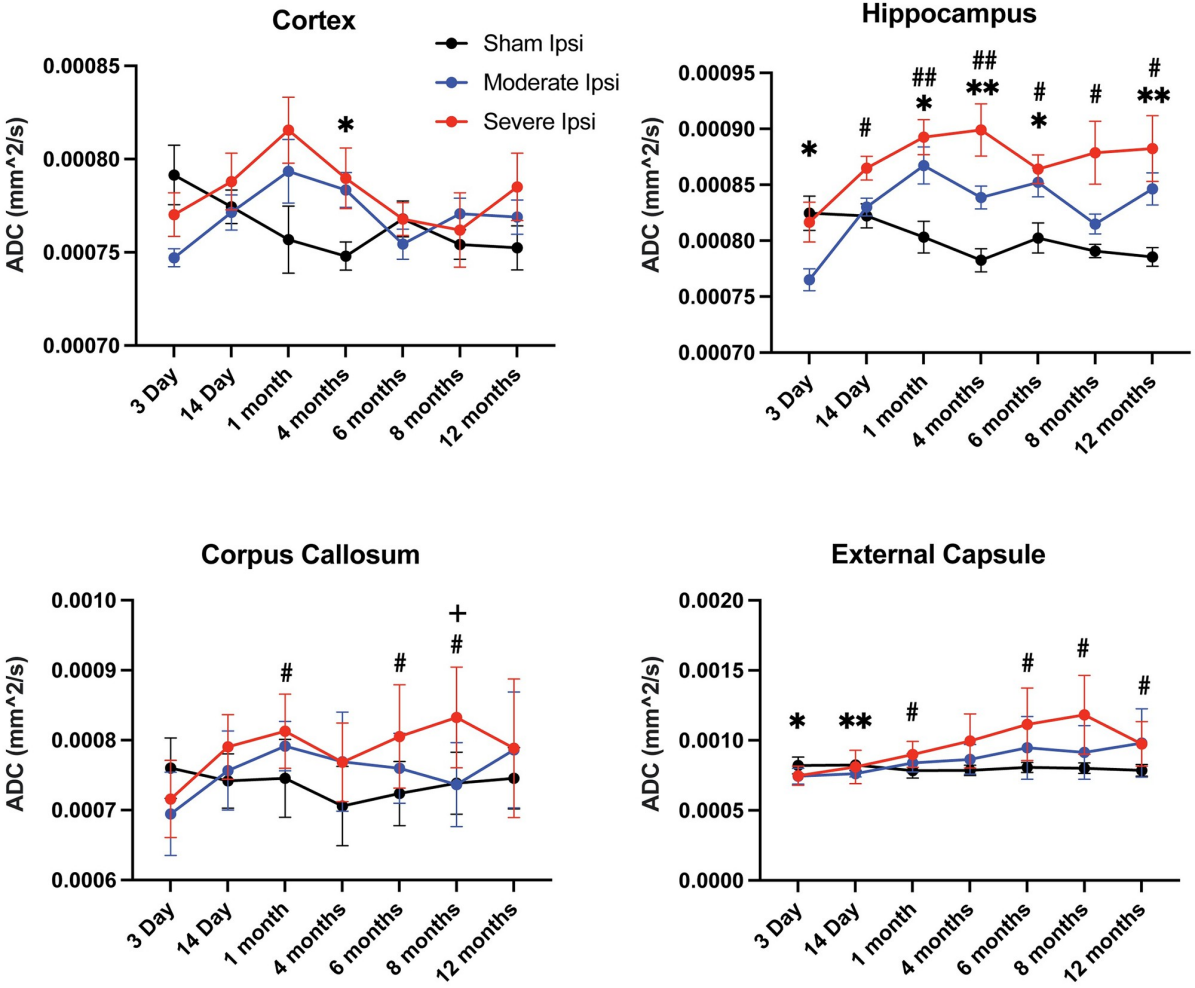
Apparent diffusion coefficient (ADC) was significantly altered in all ipsilateral ROIs. Within the cortex, significance was observed at a 4-month time point between sham and moderate animals. ADC within the ipsilateral hippocampus was significant at all post-injury time points between sham and injured groups (see S1 Table for values and groups exhibiting significance). ADC in the corpus callosum was primarily statistically significant between sham and severe groups; however, at 8 months, significant differences were also observed between moderate and severe animals. The external capsule exhibited statistically different ADC at all, but a 4-month time point between both shams versus moderate and sham versus severe groups. *p ≤ 0.05; **p ≤ 0.01.

DTI metrics were assessed in the cortex, hippocampus, corpus callosum, and external capsule ROIs on the ipsilateral side where the injury was generated. Several quantitative measurements were obtained, including ADC, FA, and RD. In measuring ADC across ROIs and injury groups, we observed an unexpected dip in values at an acute 3-day time point, reaching significance between sham and moderately injured animals in the hippocampus and external capsule (p = 0.0134; DF 14.72 and p = 0.0332; DF 15.48, respectively) (Fig 2). In the ipsilateral hippocampus, ADC values acquired at 3 days post moderate TBI were significantly disparate from ADC values at virtually all subsequent imaged time points in this animal group. As time progressed, ADC increased and remained elevated throughout the imaging, with a high incidence of significance seen in the ipsilateral hippocampus and external capsule between both sham versus moderate and sham versus severe injury groups. At 8 months post TBI, severely injured animals had significantly elevated ADC values relative to moderately injured groups in the corpus callosum white matter tract (see S1 Table for all corresponding statistical measurements and exact p-values).

**Fig 3:**
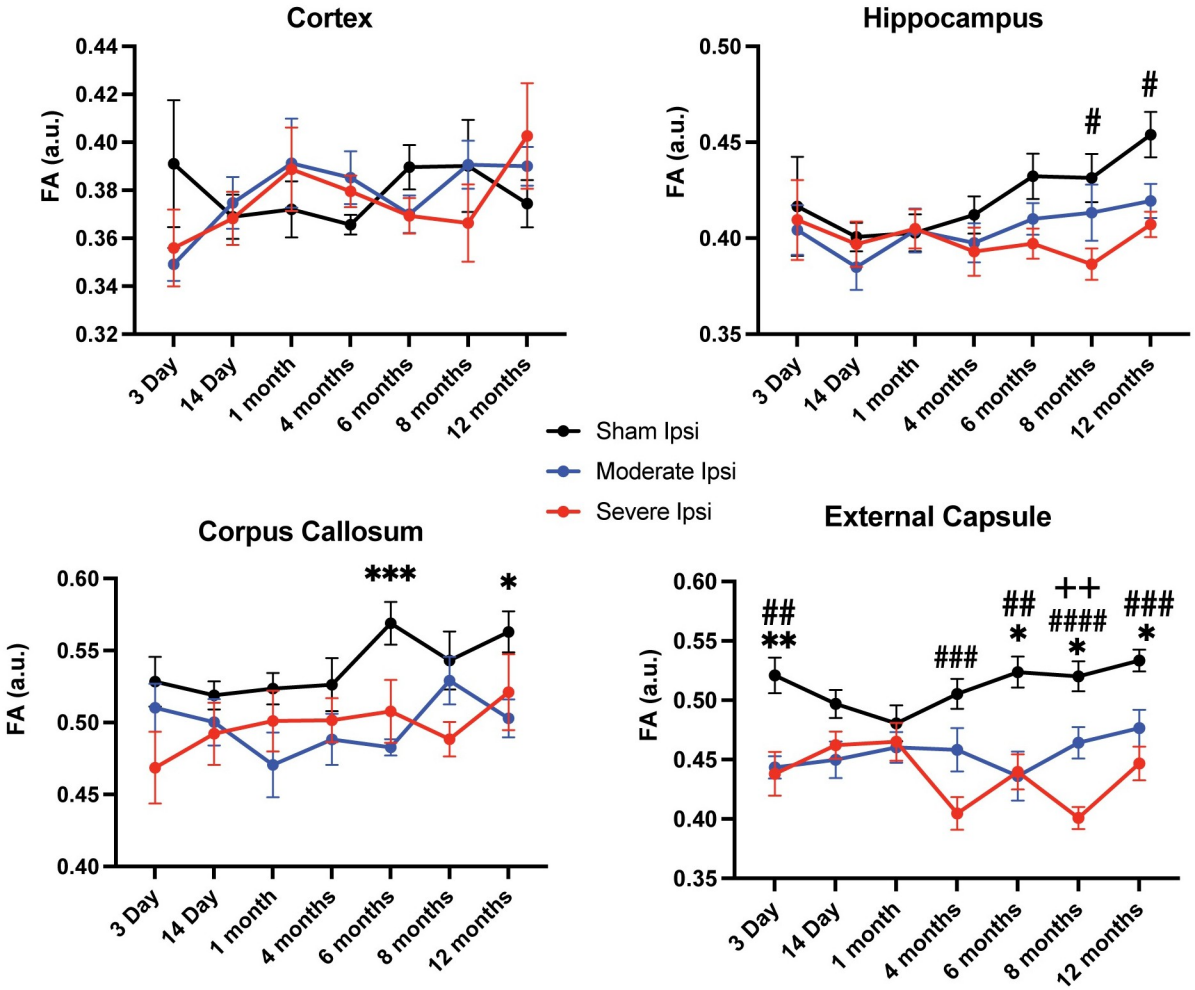
Fractional anisotropy (FA) values were significantly reduced in the ipsilateral hippocampus, corpus callosum, and external capsule. FA in the cortex initially dropped and then increased by 1 month, however, these changes were not significantly different from sham. FA values in the hippocampus were aberrantly reduced after severe injury at chronic 8- and 12-month time points. Chronic FA differences were also evident in the corpus callosum, but only between sham and moderate groups. In the external capsule, FA was reduced at several acute and chronic time points between sham and injury groups and between moderate and severe groups at 8 months post-TBI. *p ≤ 0.05; **p ≤ 0.01; ***p≤ 0.001; ****p≤ 0.0001.

As mentioned previously, FA measures fiber directionality and can be interpreted as an indicator of axonal dysfunction [36, 56]. In the present study, we did not observe any significant changes in the cortex at any time point compared to control, however there were significant differences between moderate, severe, and sham groups in all other ROIs, primarily in the ipsilateral external capsule (Fig 3). Furthermore, most of the significant FA reduction was evident at more chronic time points after injury, except for in the external capsule in which there were significant acute reductions among moderate and severe groups versus sham animals. The hippocampus, in which ADC alterations were highly significant across all time points, was only moderately affected in FA assessments with significant differences only observed between sham and severe FPI groups at two time points.

**Fig 4:**
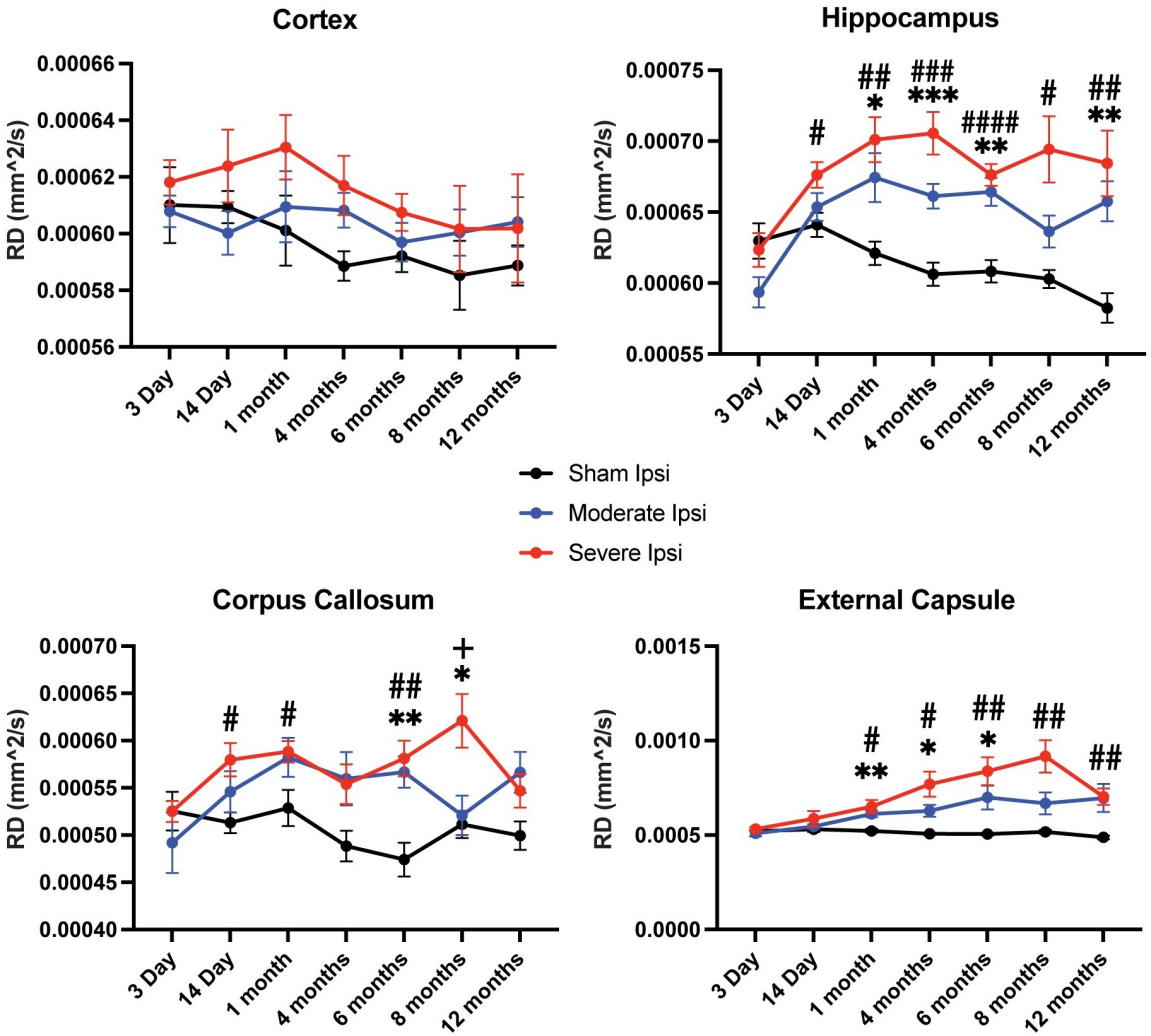
Radial diffusivity (RD) values were significantly elevated in the ipsilateral hippocampus, corpus callosum, and external capsule. Similar to what was observed in FA, RD values recorded in ipsilateral ROIs exhibited statistically significant increases compared to control groups except for the cortex. Within the hippocampus, RD was pathologically elevated at every time point between sham and injury groups except for at an acute phase 3 days post TBI. In the corpus callosum, there was statistically abnormal heightened RD in the majority of time points between sham and injured animals, while imaging at 8 months revealed a significant increase between moderate and severe RD values. The external capsule had elevated RD from 1 month until the 12-month endpoint between sham and TBI groups. *p ≤ 0.05; **p ≤ 0.01; ***p ≤ 0.001; ****p≤ 0.0001.

We also acquired RD metrics, a DTI assessment of the perpendicularity of fibers and of white matter functionality, to further evaluate microstructural alterations in white matter between uninjured and injured groups across time points. We observed that RD values resembled those of FA in that there were significant pathological elevations after both moderate and severe injury relative to sham controls in all ROIs except for the cortex (Fig 4). Analysis of this parameter showed the greatest significance in the hippocampus, followed by external capsule and corpus callosum in both subacute and chronic time points.

Taken together, our DTI assessments revealed that 1) ADC was significantly and bidirectionally altered in all ipsilateral ROIs evaluated between sham and injured groups; 2) FA values were significantly reduced in ipsilateral hippocampus and corpus callosum chronically, while reduced in the external capsule at both acute and chronic time points; 3) RD values were significantly aberrantly elevated in ipsilateral hippocampus and in major white matter tracts at the majority of time points except for 3 days post TBI; 4) DTI differences among groups were primarily observed between sham versus moderate and sham versus severe groups, however at 8 months post injury, all DTI acquisitions revealed significant differences between moderate TBI and severe TBI groups. We did not observe any significant differences in contralateral ROIs among groups.

### CBF changes after moderate and severe TBI

**Fig 5:**
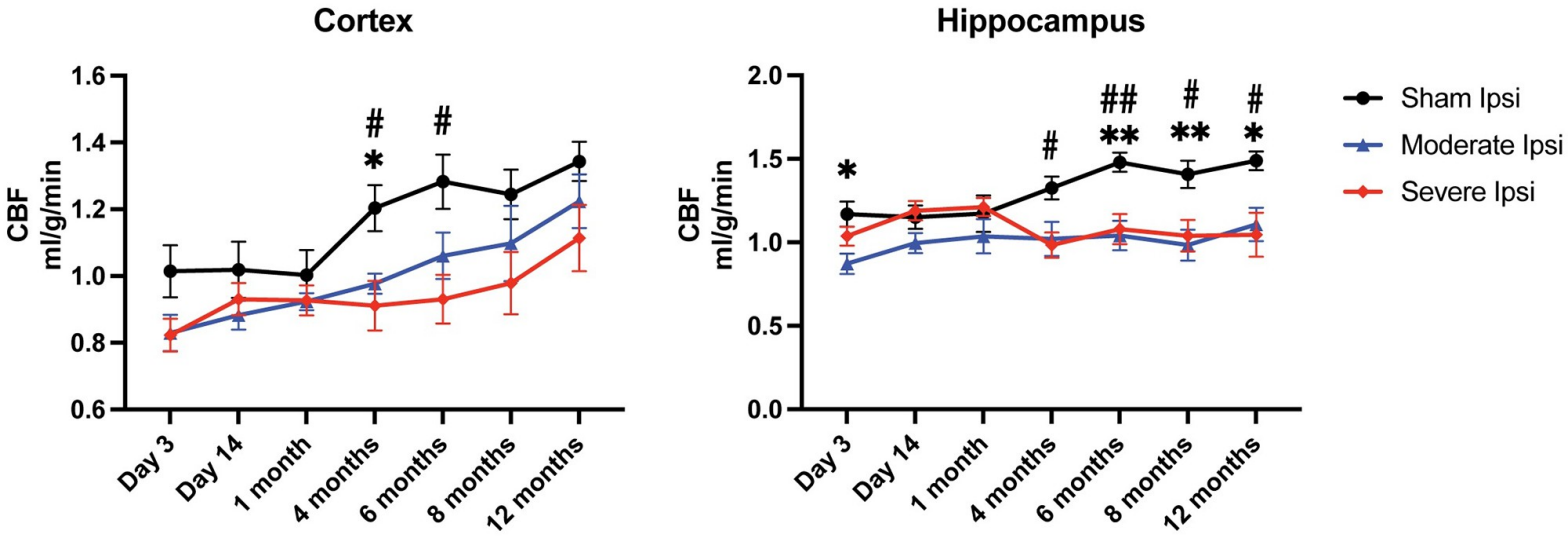
After moderate and severe TBI, PWI revealed CBF was significantly decreased in the ipsilateral cortex and hippocampus at several time points relative to sham animals. In the cortex, depressed CBF was observed at 4 and 6 months between sham versus moderate and sham versus severe groups, while the hippocampus showed regional CBF decreases at an acute 3-day time point, and chronically from 4 months out until 12 months post moderate and severe TBI relative to sham uninjured controls. *p ≤ 0.05; **p ≤ 0.01.

In addition to DTI, CBF values were also acquired from all groups and across all time points using PWI (S1 Table). We detected a perceptible drop in CBF levels in the cortex across all time points in both injury groups, reaching significance among sham versus moderate and severe at 4 months post injury, and between sham and severe at 6 months (Fig 5). In the hippocampus, an acute 3 day decrease in blood flow was recorded between sham and moderately injured animals. This acute reduction was followed by signficant decreases in blood flow at more chronic time points out until 12 months. There were no statistically significant CBF differences between moderate and severe TBI groups at any time point nor within contralateral ROIs.

### Chronic histopathology

**Fig 6:**
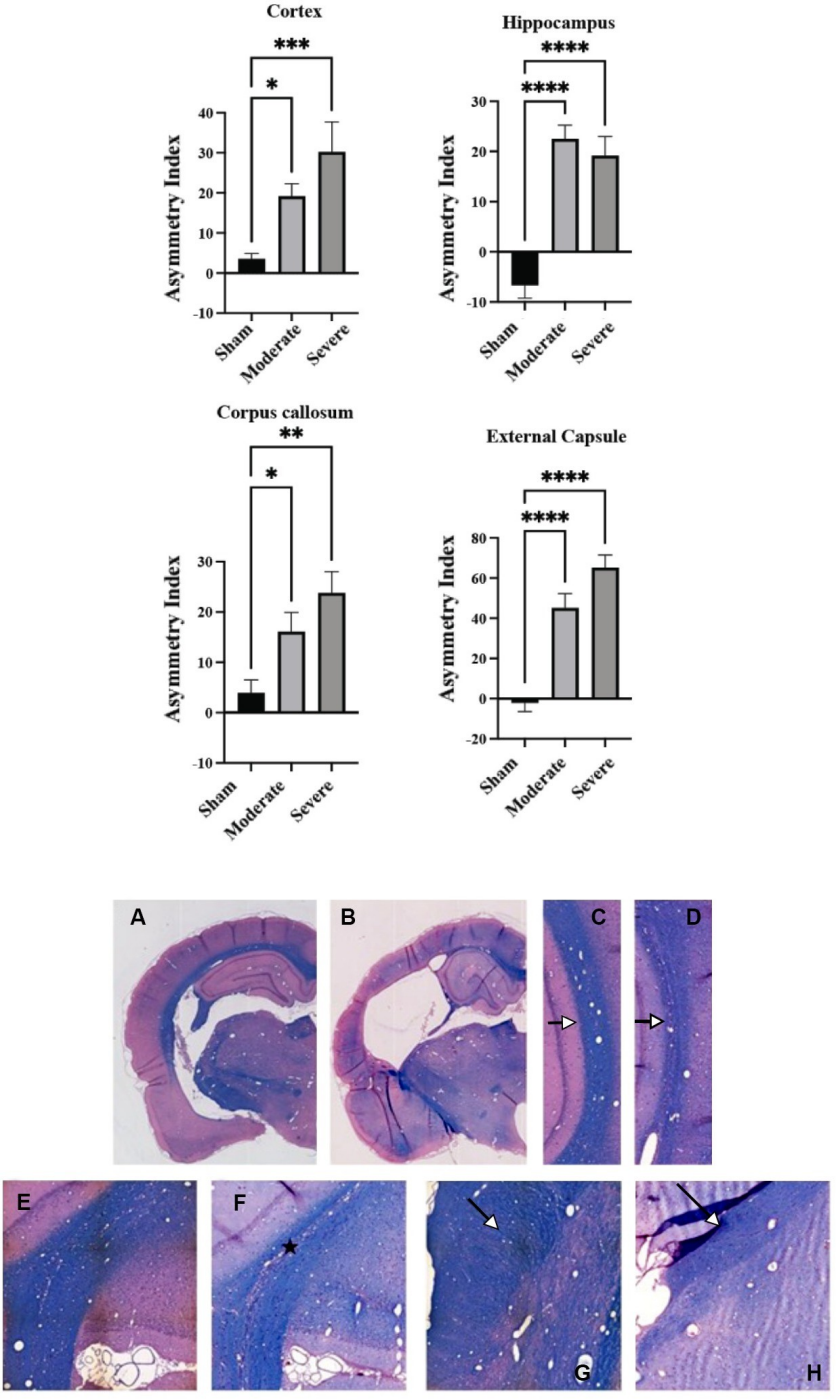
Significant histopathology was observed 12 months after moderate and severe TBI. **(Top)** Volumetric analyses revealed that both moderate and severely injured animals had significant atrophy in all ipsilateral ROIs compared to sham uninjured control animals at a 12-month chronic time point. There were no significant differences in atrophy between moderate and severe injuries. *p ≤ 0.05; **p ≤ 0.01; ***p ≤ 0.001; **** P ≤ 0.000. **(Bottom)** Double-stained hematoxylin-eosin and Luxol fast blue sections 1 year after moderate TBI or sham procedure. (**A)** Representative sham-operated animal appeared unremarkable. **(B)** Representative moderate TBI animal showed gross atrophy with a marked expansion of the ipsilateral lateral ventricle. (**C)** Higher magnification of external capsule in sham animal appeared normal. **(D)** Higher magnification of external capsule thinning after moderate TBI (arrows). **(E)** Representative sham animal showed normally stained white matter fibers. (F) Representative moderate TBI animal showed loss of white matter staining in the corpus callosum, indicative of demyelination (*). (**G)** Representative sham animal had an unremarkable cerebral peduncle. (**H)** In contrast, moderate TBI animal demonstrated atrophic changes within the ipsilateral cerebral peduncle (arrows).

At 12 months post moderate, severe, or sham surgery, all animals were sacrificed and assessed in order to detect the extent of cerebral atrophy. An asymmetry index was calculated based on structural volumes assessing atrophy between ipsilateral and contralateral sides. The greater value of the asymmetry index, the greater the degree of atrophic difference between the injured and uninjured hemispheres within each animal. Volumetric analyses of the posttraumatic atrophic cytoarchitecture revealed high levels of statistical significance between ipsilateral and contralateral ROIs in both moderate and severe injured animals relative to sham animals (Fig 6). Twelve-month histological assessments showed no differences in asymmetry indices between moderate and severe animals.

### Can DTI and PWI modalities be used as prognostic indicators?

#### Changes in the cerebral cortex

**Fig 7:**
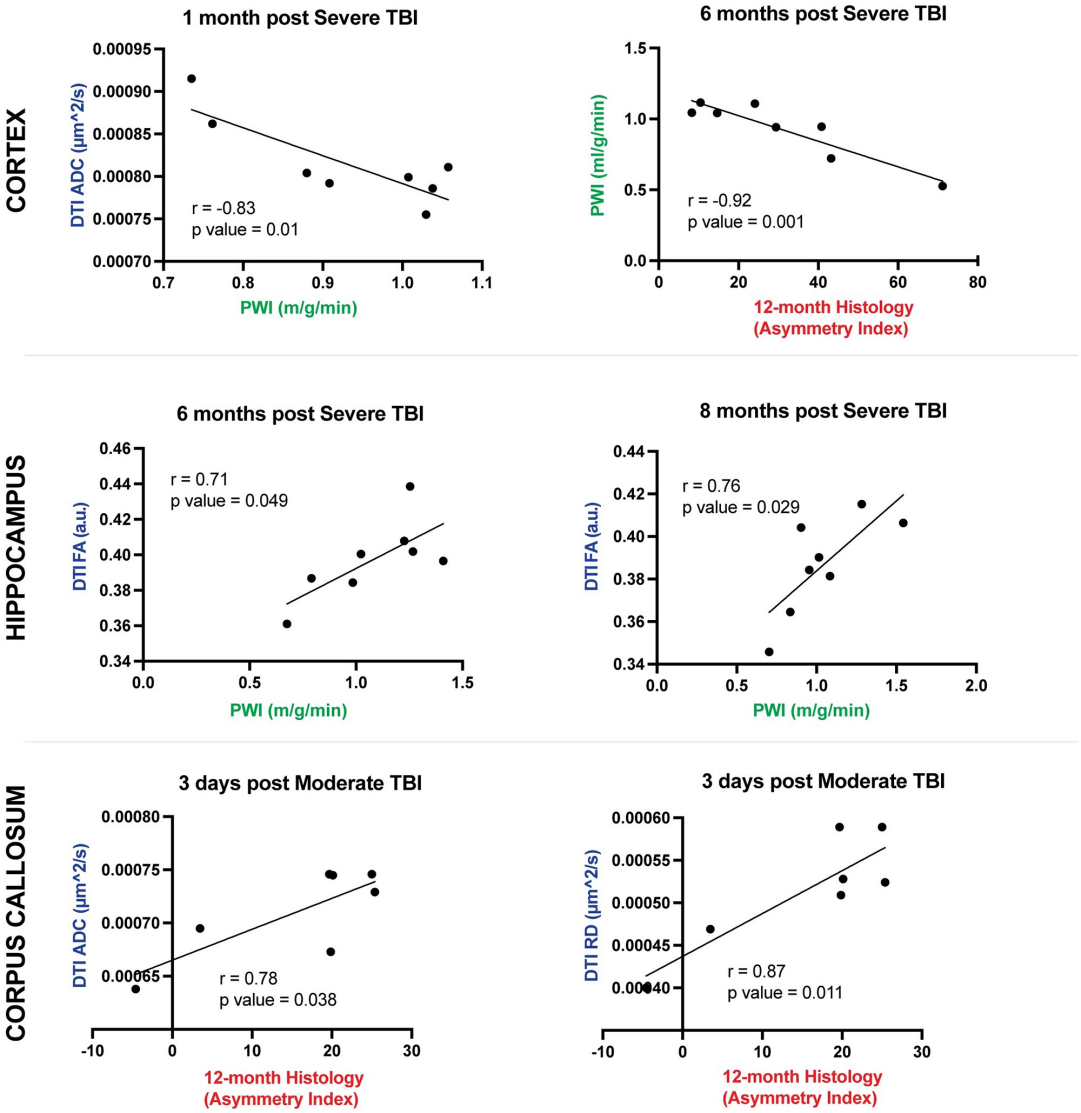
DTI and PWI modalities as prognostic indicators after moderate and severe TBI. **(Top row)** At one month after severe TBI, there was a significant correlation between ADC and PWI in the ipsilateral cortex. Lower, more normalized ADC values correlated to greater CBF perfusion. Six months after severe injury, reduced CBF perfusion in the ipsilateral cortex significantly correlated with worsened degree of chronic cortical atrophy at 12 months. **(Middle row)** Correlation analyses showed that at 6 and 8 months after severe TBI, there was a positive correlation between FA values and PWI CBF. The larger the FA value, more closely resembling sham uninjured levels, the greater the degree of CBF perfusion in the ipsilateral hippocampus. **(Bottom row)** At an acute 3-day time point, ADC and RD were noticeably reduced in the ipsilateral corpus callosum of moderate TBI animals. We observed a significant positive correlation in which the greater the value of ADC and RD, the worse the extent of atrophic histopathology after moderate TBI. These findings illustrated that the degree of DTI reduction at 3 days after moderate injury was significantly correlative of 12-month cytoprotection. Statistical details for correlation analyses can be found in the Materials and Methods section.

As shown in Fig 2, one month after severe TBI, ADC levels were aberrantly elevated in all structures, including within the cortex, consistent with expected pathological fluctuation patterns. Correlation analyses were conducted which revealed that lower ADC values were associated with greater CBF perfusion in cortical tissue at a one-month time point **(**Fig 7, **top left**). Additionally, as shown in Fig 5, we detected a significant difference in PWI between sham and severely injured animals at a 6-month time point. Using correlation analysis, we found that statistically-significant decreased CBF at 6 months negatively correlated with worsened histopathology (Fig 7, **top right**) indicative that reduced blood flow at 6 months was associated with increased cortical atrophy at 12 months after severe TBI.

#### Changes in the hippocampus

In severe groups, we found that at 6- and 8-month chronic time points, FA DTI values positively correlated with PWI CBF in ipsilateral hippocampal tissue (Fig 7, **middle row)**. This indicates that at these times points, worsened FA outcomes in terms of reduction relative to sham controls correlated with worsened CBF perfusion in the hippocampus after severe TBI. DTI and CBF correlation was not observed in moderate animals.

#### Changes in the corpus callosum

In the corpus callosum, we observed that acute ADC and RD DTI parameters positively correlated with worsened histopathology at 12 months (Fig 7, **bottom row**). Beginning approximately 14 days to 1 month post TBI, ADC and RD values are significantly aberrantly elevated, however at 3 days after injury, we observed a clear reduction in these values. The observed correlation indicates that the less the initial decrease in these values, the greater the ipsilateral atrophy after moderate TBI was present. This unexpected correlation was not observed after severe TBI.

## Discussion

In the current study, we evaluated how DTI and CBF PWI measurements evolved over time in a preclinical model of moderate and severe TBI, as well as the degree of chronic cerebral atrophy at 1 year after injury. Furthermore, we sought to establish whether there was a significant correlative relationship between MRI parameters and 12-month histopathology at various time points over the course of the study.

Consistent with the clinical literature, we detected DTI and PWI alterations that reflected injury-induced aberrations observed in TBI patients [57]. In all structures analyzed, we found that ADC dropped at an acute 3-day time point, reaching significance between moderate and sham groups in both the hippocampus and external capsule. These ADC changes are consistent with experimental clinical findings in which axial diffusivity is reported to be significantly reduced at acute time points in patients, potentially related to persistent axonal injury, demyelination, and vasogenic and cytotoxic edema [33]. In terms of the injury model, previous studies have documented temporal and regionally specific structural changes in gray and white matter structures including BBB, axonal damage and visually edematous appearing tissue [40, 42, 51, 58]. We further speculate that acute ADC decreases could be due to a reduction of the extracellular space secondary to injury-associated ischemic changes. Blood flow studies have shown that moderate and severe FPI produced ischemic levels in areas of the evolving contusion [41, 43]. In this regard, Shen and colleagues reported in acute stroke patients a hyperacute reduction in ADC values that was attributed to the observed slowdown of water molecules by cellular edema post-infarction [35]. The mechanisms related to cytotoxic edema are often associated with ischemia-mediated disruption of metabolism and loss of ionic gradients, which can increase the flow of water from the extracellular space into intracellular compartments. Additional factors can also contribute to a reduction in water molecule diffusions, such as the subsequent decrease of extracellular space and increased viscosity [59]. Beginning at 14 days to 1 month, we observed that ADC began to increase within all structures analyzed after both moderate and severe TBI and remained elevated throughout the duration of the imaging. These findings are consistent with microstructural changes after TBI in which an increase in water diffusion is associated with loss of neuronal tissue, cavitation, and injury-induced gliosis [60].

In the present study, we found a significant reduction in FA values in the ipsilateral hippocampus, corpus callosum, and external capsule after moderate and severe TBI. The cortex demonstrated biphasic FA changes. FA reduction implies axonal pathology, and it has been reported that increased FA values after the injury is associated with better outcomes and reparative reorganization of axonal fibers [61]. Yuh et al. reported that severely reduced FA values after TBI in patients presenting with intracranial lesions were a significant predictor of poor 3- and 6-month GOS-E outcome measures, and superior to conventional imaging and demographic/socioeconomic characteristics [62].

Radial diffusivity (RD) was the third DTI metric we assessed longitudinally after TBI. Our studies have documented patterns of axonal damage using β-APP [51]. We observed significant pathological elevations at a majority of imaging time points in the hippocampus and in major white matter tracts corpus callosum and external capsule. Our findings showed significantly increased RD in 3 of 4 structures across the majority of time points. Importantly, this pathological increase has been shown to be associated with the development and severity of post-concussive syndrome [63]. In another study, Perez and colleagues showed that increased RD values after traumatic axonal injury (TAI) associated brain injury was related to the degree of injury severity, while parallel diffusivity was an indicator of neuronal connectivity speed and axonal tract integrity. Furthermore, increased RD may be a contributing factor to reduced FA values [64].

The study used PWI to measure cerebral blood flow (CBF) between injured and non-injured ipsilateral cortices and hippocampi across time points. We observed decreased CBF at virtually all time points, reaching significance most frequently in chronic periods. We did not observe progressive changes in gray matter CBF after a 4-month time point, indicating that injury-induced blood flow reduction was sustained out to one year, the end point of the present study–which is consistent with published reports on patients with moderate to severe TBI [65]. When CBF is pathologically reduced, there is disruption of metabolic processes and a mismatch between supply and demand for oxygen and glucose, leading to cell death, long-term impairment, and dysfunction [47, 66–69]. There is a significant positive correlation between injury-induced lowered CBF and cognitive deficits, even in mild TBI [70, 71]. Previous studies have shown that a reduction in CBF correlates with reduction in ADC, signifying ischemia-related changes [72], which, notably, is consistent with our findings at 1 month post severe TBI in the cortex.

In addition to reporting longitudinal DTI and PWI changes across 7 acute and chronic time points after moderate and severe TBI, we also analyzed the degree of cerebral atrophy at 1 year post TBI. As mentioned previously, there was highly significant asymmetry between ipsilateral and contralateral volumes in all structures evaluated at this chronic timepoint, which is consistent with the literature [58, 73]. The degree of atrophy trended in an injury-severity dependent manner in all ROIs except for the hippocampus.

Given the copious amount of information provided using multiple MRI modalities, an important goal of this study was to determine whether there were significant correlations between measurements and the degree to which these modalities could be utilized as prognostic indicators of long-term outcomes. In the present study, we found that chronic DTI and PWI values correlated significantly after severe TBI in the cortex and hippocampus. Furthermore, we determined that the greater the aberrancy of ADC and FA measurements, the greater the pathological reductions in CBF. Thus, ADC and FA values could be used to predict the magnitude of diminished perfusion in the severely injured cortex and hippocampus, and vice versa.

We also found that, at a chronic 6-month time point, the extent of decreased CBF was indicative of the magnitude of cortical atrophy at 12 months. A direct and significant correlation (p-value = 0.001) between these two variables signified that a loss of CBF perfusion is suggestive of patterns of worsened histopathological outcomes [41]. Causality, however, for example whether decreases in CBF contributed to atrophic tissue progression, cannot be addressed in the current study.

As shown and discussed above, ADC and RD measurements are significantly and pathologically elevated after TBI across time points, except in the acute 3-day phase in which these metrics drop below sham levels. Interestingly, we found that the degree of this drop in ADC and RD values measured in the corpus callosum may be indicative of some endogenous protective mechanism that results in lessened histopathology at 12 months. Further investigation of contributing factors is warranted.

In the present study assessing MRI modalities in moderate and severe FPI, we did not observe extensive significance between injury severity groups. At 8 months, we found that in all three DTI metrics assessed, severe TBI animals had greater diffusion abnormalities in major white matter tracts relative to moderately injured animals. In our preclinical fluid-percussion TBI model, a moderate injury pulse ranges between 1.8 and 2.2 ATM while severe TBI is defined between by 2.3 to 2.5 [41, 51]. In the present study, the average moderate injury ATM was 1.99 and severe animals averaged 2.3 ATM. We speculate that perhaps the pressure pulse was not disparate enough to cause multiple significant MRI alterations between injury groups, except at an 8-month time point. Why injury severity differences were only evident at this specific posttraumatic time point remains to be determined. Given the progressive nature of TBI, it is possible that this chronic time point captured the peak of diffusion abnormalities in major white matter tracts, and by 12 months, endogenous protective mechanisms are sufficiently robust enough to resolve a portion of the remaining white matter dysfunction in severely injured animals. It would be valuable to assess MRI metrics of differing degrees of injury severity in additional models of TBI, such as in closed cortical impact or in concussive weight drop TBI models. An additional consideration for this study would be to increase the number of animals per group although our sample size analysis indicated that 8–10 animals per groups would be sufficient for detecting a significant difference.

There are inherent limitations to using DTI for evaluating comparative changes in microstructural anisotropy. Averaged DTI voxels on the scale of MRI measurements have limited precision in differentiating changes in white matter fiber orientation, especially in longer-range or highly complex tracts [74]. Furthermore, there is much heterogenicity when assessing DTI metrics in clinical populations, and thus it can be challenging to draw conclusions based on DTI values alone. That being said, Hulkower et al., in a comprehensive review, showed that aberrant reductions in FA values, which are the most frequently reported DTI metric across studies, were positively correlated to attention, executive function, and memory impairment [75]. However, conflicting reports are not uncommon, underscoring the widespread and inherent heterogeneity of brain injury and DTI measurements [76]. Given the varying degrees of diffusion abnormalities we observed across ADC, FA, and RD in all ROIs (i.e. in the ipsilateral hippocampus, FA was only significant at 8 and 12 months while ADC values were significantly different at every assessed time point), we emphasize the importance of using multiple DTI metrics to achieve a more accurate and precise understanding of the extent of the injury-induced disruption of white matter integrity.

Female rats were not utilized in the present study as hormonal fluctuations with the female reproductive cycle can introduce biological variability. Whether MRI patterns, histological findings, and correlative outcomes are applicable to TBI in female rats remain to be determined as gender is a significant biological variable. Furthermore, repeated exposure to gaseous anesthetic, such as what was conducted at each MRI session, could potentially cause unanticipated changes unrelated to TBI. However, at levels administered, we do not anticipate significant effects on outcomes.

In this study, we demonstrated that non-invasive MRI provided valuable information for identifying microstructural abnormalities in the brain after TBI. Utilizing longitudinal imaging out to one year after moderate and severe injury allowed for the visualization of how DTI and PWI metrics vary over time. We also identified statistically significant correlations among MRI modalities as well as to chronic histopathological atrophy as a result of the progressive nature of the injury in both moderate and severe TBI.

The temporal and regional changes using a multimodal analytical approach offers a powerful strategy to identify therapeutic targets. Recognizing these alterations and identifying various windows for intervention allows for the potential of MRI modalities as essential long-term predictors of TBI-induced sequelae and for implementation of time-targeted treatment therapies in the clinical setting.

## Supporting information

S1 Table Statistical values for DTI and PWI. Data presented in this table represent the significant differences between groups and time points for ipsilateral DTI and PWI measurements.
(XLSX)

S2 Table Full linear regression statistics. Limited correlations were presented in the manuscript and this table reports the full regression analysis for the study.
(XLSX)

## References

[1] Hyder AA, Wunderlich CA, Puvanachandra P, Gururaj G, Kobusingye OC. The impact of traumatic brain injuries: a global perspective. NeuroRehabilitation. 2007;22(5):341–53. 18162698

[2] Taylor CA, Bell JM, Breiding MJ, Xu L. Traumatic Brain Injury-Related Emergency Department Visits, Hospitalizations, and Deaths—United States, 2007 and 2013. Morbidity and mortality weekly report Surveillance summaries (Washington, DC: 2002). 2017;66(9):1–16. doi: 10.15585/mmwr.ss6609a1 28301451 PMC5829835

[3] CDC. QuickStats: Injury and traumatic brain injury-related death rates by age—United States M.

[4] Ramos-Cejudo J, Wisniewski T, Marmar C, Zetterberg H, Blennow K, de Leon MJ, et al. Traumatic Brain Injury and Alzheimer’s Disease: The Cerebrovascular Link. EBioMedicine. 2018;28:21–30. doi: 10.1016/j.ebiom.2018.01.021 29396300 PMC5835563

[5] Sanders MJ, Dietrich WD, Green EJ. Cognitive function following traumatic brain injury: effects of injury severity and recovery period in a parasagittal fluid-percussive injury model. J Neurotrauma. 1999;16(10):915–25. doi: 10.1089/neu.1999.16.915 10547100

[6] Bramlett HM, Green EJ, Dietrich WD. Hippocampally dependent and independent chronic spatial navigational deficits following parasagittal fluid percussion brain injury in the rat. Brain Res. 1997;762(1–2):195–202. doi: 10.1016/s0006-8993(97)00387-9 9262173

[7] Mohamed AZ, Nestor PJ, Cumming P, Nasrallah FA, Alzheimer’s Disease Neuroimaging I. Traumatic brain injury fast-forwards Alzheimer’s pathology: evidence from amyloid positron emission tomorgraphy imaging. J Neurol. 2022;269(2):873–84. doi: 10.1007/s00415-021-10669-5 34191080

[8] Harris TC, de Rooij R, Kuhl E. The Shrinking Brain: Cerebral Atrophy Following Traumatic Brain Injury. Ann Biomed Eng. 2019;47(9):1941–59. doi: 10.1007/s10439-018-02148-2 30341741 PMC6757025

[9] Johnson NH, Kerr NA, de Rivero Vaccari JP, Bramlett HM, Keane RW, Dietrich WD. Genetic predisposition to Alzheimer’s disease alters inflammasome activity after traumatic brain injury. Transl Res. 2023. doi: 10.1016/j.trsl.2023.02.001 36758791 PMC10192027

[10] Graham NSN, Jolly A, Zimmerman K, Bourke NJ, Scott G, Cole JH, et al. Diffuse axonal injury predicts neurodegeneration after moderate-severe traumatic brain injury. Brain. 2020;143(12):3685–98. doi: 10.1093/brain/awaa316 33099608

[11] Bramlett HM, Dietrich WD. Long-Term Consequences of Traumatic Brain Injury: Current Status of Potential Mechanisms of Injury and Neurological Outcomes. J Neurotrauma. 2015;32(23):1834–48. doi: 10.1089/neu.2014.3352 25158206 PMC4677116

[12] Bramlett HM, Dietrich WD, Green EJ, Busto R. Chronic histopathological consequences of fluid-percussion brain injury in rats: effects of post-traumatic hypothermia. Acta Neuropathol. 1997;93(2):190–9. doi: 10.1007/s004010050602 9039468

[13] Blatter DD, Bigler ED, Gale SD, Johnson SC, Anderson CV, Burnett BM, et al. MR-based brain and cerebrospinal fluid measurement after traumatic brain injury: correlation with neuropsychological outcome. AJNR American journal of neuroradiology. 1997;18(1):1–10. 9010514 PMC8337869

[14] Hofman PA, Stapert SZ, van Kroonenburgh MJ, Jolles J, de Kruijk J, Wilmink JT. MR imaging, single-photon emission CT, and neurocognitive performance after mild traumatic brain injury. AJNR American journal of neuroradiology. 2001;22(3):441–9. 11237964 PMC7976823

[15] Trivedi MA, Ward MA, Hess TM, Gale SD, Dempsey RJ, Rowley HA, et al. Longitudinal changes in global brain volume between 79 and 409 days after traumatic brain injury: relationship with duration of coma. J Neurotrauma. 2007;24(5):766–71. doi: 10.1089/neu.2006.0205 17518532 PMC2627781

[16] Wilde EA, Bigler ED, Pedroza C, Ryser DK. Post-traumatic amnesia predicts long-term cerebral atrophy in traumatic brain injury. Brain Inj. 2006;20(7):695–9. doi: 10.1080/02699050600744079 16809201

[17] Himanen L, Portin R, Isoniemi H, Helenius H, Kurki T, Tenovuo O. Cognitive functions in relation to MRI findings 30 years after traumatic brain injury. Brain Inj. 2005;19(2):93–100. doi: 10.1080/02699050410001720031 15841753

[18] Tomaiuolo F, Worsley KJ, Lerch J, Di Paola M, Carlesimo GA, Bonanni R, et al. Changes in white matter in long-term survivors of severe non-missile traumatic brain injury: a computational analysis of magnetic resonance images. J Neurotrauma. 2005;22(1):76–82. doi: 10.1089/neu.2005.22.76 15665603

[19] Bigler ED. Anterior and middle cranial fossa in traumatic brain injury: relevant neuroanatomy and neuropathology in the study of neuropsychological outcome. Neuropsychology. 2007;21(5):515–31. doi: 10.1037/0894-4105.21.5.515 17784800

[20] Povlishock JT, Becker DP, Cheng CL, Vaughan GW. Axonal change in minor head injury. J Neuropathol Exp Neurol. 1983;42(3):225–42. doi: 10.1097/00005072-198305000-00002 6188807

[21] Bigler ED, Maxwell WL. Neuroimaging and neuropathology of TBI. NeuroRehabilitation. 2011;28(2):63–74. doi: 10.3233/NRE-2011-0633 21447905

[22] Maas AI, Stocchetti N, Bullock R. Moderate and severe traumatic brain injury in adults. The Lancet Neurology. 2008;7(8):728–41. doi: 10.1016/S1474-4422(08)70164-9 18635021

[23] Wilde EA, Ramos MA, Yallampalli R, Bigler ED, McCauley SR, Chu Z, et al. Diffusion tensor imaging of the cingulum bundle in children after traumatic brain injury. Dev Neuropsychol. 2010;35(3):333–51. doi: 10.1080/87565641003696940 20446136 PMC3229222

[24] Johnson VE, Stewart W, Smith DH. Axonal pathology in traumatic brain injury. Exp Neurol. 2013;246:35–43. doi: 10.1016/j.expneurol.2012.01.013 22285252 PMC3979341

[25] Mutch CA, Talbott JF, Gean A. Imaging Evaluation of Acute Traumatic Brain Injury. Neurosurg Clin N Am. 2016;27(4):409–39. doi: 10.1016/j.nec.2016.05.011 27637393 PMC5027071

[26] Shenton ME, Hamoda HM, Schneiderman JS, Bouix S, Pasternak O, Rathi Y, et al. A review of magnetic resonance imaging and diffusion tensor imaging findings in mild traumatic brain injury. Brain Imaging Behav. 2012;6(2):137–92. doi: 10.1007/s11682-012-9156-5 22438191 PMC3803157

[27] Haber M, Amyot F, Lynch CE, Sandsmark DK, Kenney K, Werner JK, et al. Imaging biomarkers of vascular and axonal injury are spatially distinct in chronic traumatic brain injury. J Cereb Blood Flow Metab. 2021;41(8):1924–38. doi: 10.1177/0271678X20985156 33444092 PMC8327117

[28] Lee H, Wintermark M, Gean AD, Ghajar J, Manley GT, Mukherjee P. Focal lesions in acute mild traumatic brain injury and neurocognitive outcome: CT versus 3T MRI. J Neurotrauma. 2008;25(9):1049–56. doi: 10.1089/neu.2008.0566 18707244

[29] Niogi SN, Mukherjee P, Ghajar J, Johnson C, Kolster RA, Sarkar R, et al. Extent of microstructural white matter injury in postconcussive syndrome correlates with impaired cognitive reaction time: a 3T diffusion tensor imaging study of mild traumatic brain injury. AJNR American journal of neuroradiology. 2008;29(5):967–73. doi: 10.3174/ajnr.A0970 18272556 PMC8128563

[30] Kim JJ, Gean AD. Imaging for the diagnosis and management of traumatic brain injury. Neurotherapeutics. 2011;8(1):39–53. doi: 10.1007/s13311-010-0003-3 21274684 PMC3026928

[31] Skandsen T, Kvistad KA, Solheim O, Strand IH, Folvik M, Vik A. Prevalence and impact of diffuse axonal injury in patients with moderate and severe head injury: a cohort study of early magnetic resonance imaging findings and 1-year outcome. J Neurosurg. 2010;113(3):556–63. doi: 10.3171/2009.9.JNS09626 19852541

[32] Wallace EJ, Mathias JL, Ward L. Diffusion tensor imaging changes following mild, moderate and severe adult traumatic brain injury: a meta-analysis. Brain Imaging Behav. 2018;12(6):1607–21. doi: 10.1007/s11682-018-9823-2 29383621

[33] Mac Donald CL, Dikranian K, Bayly P, Holtzman D, Brody D. Diffusion tensor imaging reliably detects experimental traumatic axonal injury and indicates approximate time of injury. J Neurosci. 2007;27(44):11869–76. doi: 10.1523/JNEUROSCI.3647-07.2007 17978027 PMC2562788

[34] Belanger HG, Vanderploeg RD, Curtiss G, Warden DL. Recent neuroimaging techniques in mild traumatic brain injury. J Neuropsychiatry Clin Neurosci. 2007;19(1):5–20. doi: 10.1176/jnp.2007.19.1.5 17308222

[35] Shen JM, Xia XW, Kang WG, Yuan JJ, Sheng L. The use of MRI apparent diffusion coefficient (ADC) in monitoring the development of brain infarction. Bmc Med Imaging. 2011;11. doi: 10.1186/1471-2342-11-2 21211049 PMC3022840

[36] Wada T, Asano Y, Shinoda J. Decreased Fractional Anisotropy Evaluated Using Tract-Based Spatial Statistics and Correlated with Cognitive Dysfunction in Patients with Mild Traumatic Brain Injury in the Chronic Stage. Am J Neuroradiol. 2012;33(11):2117–22. doi: 10.3174/ajnr.A3141 22723057 PMC7965599

[37] Sundman MH, Hall EE, Chen NK. Examining the relationship between head trauma and neurodegenerative disease: A review of epidemiology, pathology and neuroimaging techniques. J Alzheimers Dis Parkinsonism. 2014;4. doi: 10.4172/2161-0460.1000137 25324979 PMC4196712

[38] Farbota KD, Sodhi A, Bendlin BB, McLaren DG, Xu G, Rowley HA, et al. Longitudinal volumetric changes following traumatic brain injury: a tensor-based morphometry study. J Int Neuropsychol Soc. 2012;18(6):1006–18. doi: 10.1017/S1355617712000835 22883443 PMC3571099

[39] Salehi A, Zhang JH, Obenaus A. Response of the cerebral vasculature following traumatic brain injury. J Cereb Blood Flow Metab. 2017;37(7):2320–39. doi: 10.1177/0271678X17701460 28378621 PMC5531360

[40] Dietrich WD, Alonso O, Halley M. Early microvascular and neuronal consequences of traumatic brain injury: a light and electron microscopic study in rats. J Neurotrauma. 1994;11(3):289–301. doi: 10.1089/neu.1994.11.289 7996583

[41] Dietrich WD, Alonso O, Busto R, Prado R, Zhao W, Dewanjee MK, et al. Posttraumatic cerebral ischemia after fluid percussion brain injury: an autoradiographic and histopathological study in rats. Neurosurgery. 1998;43(3):585–93; discussion 93–4. doi: 10.1097/00006123-199809000-00105 9733314

[42] Bramlett HM, Green EJ, Dietrich WD. Exacerbation of cortical and hippocampal CA1 damage due to posttraumatic hypoxia following moderate fluid-percussion brain injury in rats. Journal of Neurosurgery. 1999;91(4):653–9. doi: 10.3171/jns.1999.91.4.0653 10507388

[43] Dietrich WD, Alonso O, Busto R, Prado R, Dewanjee S, Dewanjee MK, et al. Widespread hemodynamic depression and focal platelet accumulation after fluid percussion brain injury: a double-label autoradiographic study in rats. J Cereb Blood Flow Metab. 1996;16(3):481–9. doi: 10.1097/00004647-199605000-00015 8621753

[44] Kochanek PM, Hendrich KS, Dixon CE, Schiding JK, Williams DS, Ho C. Cerebral blood flow at one year after controlled cortical impact in rats: assessment by magnetic resonance imaging. J Neurotrauma. 2002;19(9):1029–37. doi: 10.1089/089771502760341947 12482116

[45] Dixon CE, Lyeth BG, Povlishock JT, Findling RL, Hamm RJ, Marmarou A, et al. A fluid percussion model of experimental brain injury in the rat. J Neurosurg. 1987;67(1):110–9. doi: 10.3171/jns.1987.67.1.0110 3598659

[46] Williams DS, Detre JA, Leigh JS, Koretsky AP. Magnetic-Resonance-Imaging of Perfusion Using Spin Inversion of Arterial Water. P Natl Acad Sci USA. 1992;89(1):212–6. doi: 10.1073/pnas.89.1.212 1729691 PMC48206

[47] Dietrich WD, Alonso O, Busto R, Ginsberg MD. Widespread metabolic depression and reduced somatosensory circuit activation following traumatic brain injury in rats. J Neurotrauma. 1994;11(6):629–40. doi: 10.1089/neu.1994.11.629 7723063

[48] Suresh K. An overview of randomization techniques: An unbiased assessment of outcome in clinical research. J Hum Reprod Sci. 2011;4(1):8–11. doi: 10.4103/0974-1208.82352 21772732 PMC3136079

[49] Agrawal R, Noble E, Vergnes L, Ying Z, Reue K, Gomez-Pinilla F. Dietary fructose aggravates the pathobiology of traumatic brain injury by influencing energy homeostasis and plasticity. J Cereb Blood Flow Metab. 2016;36(5):941–53. doi: 10.1177/0271678X15606719 26661172 PMC4853835

[50] Kinoshita K, Kraydieh S, Alonso O, Hayashi N, Dietrich WD. Effect of posttraumatic hyperglycemia on contusion volume and neutrophil accumulation after moderate fluid-percussion brain injury in rats. J Neurotrauma. 2002;19(6):681–92. doi: 10.1089/08977150260139075 12165130

[51] Bramlett HM, Kraydieh S, Green EJ, Dietrich WD. Temporal and regional patterns of axonal damage following traumatic brain injury: a beta-amyloid precursor protein immunocytochemical study in rats. J Neuropathol Exp Neurol. 1997;56(10):1132–41. doi: 10.1097/00005072-199710000-00007 9329457

[52] Narayana PA. White matter changes in patients with mild traumatic brain injury: MRI perspective. Concussion. 2017;2(2):CNC35. doi: 10.2217/cnc-2016-0028 30202576 PMC6093760

[53] Jiang H, van Zijl PC, Kim J, Pearlson GD, Mori S. DtiStudio: resource program for diffusion tensor computation and fiber bundle tracking. Comput Methods Programs Biomed. 2006;81(2):106–16. doi: 10.1016/j.cmpb.2005.08.004 16413083

[54] Williams DS, Detre JA, Leigh JS, Koretsky AP. Magnetic resonance imaging of perfusion using spin inversion of arterial water. Proc Natl Acad Sci U S A. 1992;89(1):212–6. doi: 10.1073/pnas.89.1.212 1729691 PMC48206

[55] Zilles KJ. The cortex of the rat: a stereotaxic atlas. Berlin; New York: Springer-Verlag; 1985. v, 121 p. p.

[56] Beaulieu C. The basis of anisotropic water diffusion in the nervous system—a technical review. NMR Biomed. 2002;15(7–8):435–55. doi: 10.1002/nbm.782 12489094

[57] Hu L, Yang S, Jin B, Wang C. Advanced Neuroimaging Role in Traumatic Brain Injury: A Narrative Review. Front Neurosci. 2022;16:872609. doi: 10.3389/fnins.2022.872609 35495065 PMC9043279

[58] Bramlett HM, Dietrich WD. Quantitative structural changes in white and gray matter 1 year following traumatic brain injury in rats. Acta Neuropathol. 2002;103(6):607–14. doi: 10.1007/s00401-001-0510-8 12012093

[59] Jha RM, Kochanek PM, Simard JM. Pathophysiology and treatment of cerebral edema in traumatic brain injury. Neuropharmacology. 2019;145(Pt B):230–46. doi: 10.1016/j.neuropharm.2018.08.004 30086289 PMC6309515

[60] Hutchinson EB, Schwerin SC, Avram AV, Juliano SL, Pierpaoli C. Diffusion MRI and the detection of alterations following traumatic brain injury. J Neurosci Res. 2018;96(4):612–25. doi: 10.1002/jnr.24065 28609579 PMC5729069

[61] Sidaros A, Engberg AW, Sidaros K, Liptrot MG, Herning M, Petersen P, et al. Diffusion tensor imaging during recovery from severe traumatic brain injury and relation to clinical outcome: a longitudinal study. Brain. 2008;131(Pt 2):559–72. doi: 10.1093/brain/awm294 18083753

[62] Yuh EL, Cooper SR, Mukherjee P, Yue JK, Lingsma HF, Gordon WA, et al. Diffusion tensor imaging for outcome prediction in mild traumatic brain injury: a TRACK-TBI study. J Neurotrauma. 2014;31(17):1457–77. doi: 10.1089/neu.2013.3171 24742275 PMC4144386

[63] Khong E, Odenwald N, Hashim E, Cusimano MD. Diffusion Tensor Imaging Findings in Post-Concussion Syndrome Patients after Mild Traumatic Brain Injury: A Systematic Review. Frontiers in neurology. 2016;7:156. doi: 10.3389/fneur.2016.00156 27698651 PMC5027207

[64] Perez AM, Adler J, Kulkarni N, Strain JF, Womack KB, Diaz-Arrastia R, et al. Longitudinal white matter changes after traumatic axonal injury. J Neurotrauma. 2014;31(17):1478–85. doi: 10.1089/neu.2013.3216 24738754 PMC4144365

[65] Gaggi NL, Ware JB, Dolui S, Brennan D, Torrellas J, Wang Z, et al. Temporal dynamics of cerebral blood flow during the first year after moderate-severe traumatic brain injury: A longitudinal perfusion MRI study. Neuroimage Clin. 2023;37:103344. doi: 10.1016/j.nicl.2023.103344 36804686 PMC9969322

[66] Giza CC, Hovda DA. The neurometabolic cascade of concussion. J Athl Training. 2001;36(3):228–35. 12937489 PMC155411

[67] Blaya M, Truettner J, Zhao W, Bramlett H, Dietrich WD. Mild Hyperthermia Aggravates Glucose Metabolic Consequences in Repetitive Concussion. Int J Mol Sci. 2020;21(2). doi: 10.3390/ijms21020609 31963504 PMC7013838

[68] Passineau MJ, Zhao W, Busto R, Dietrich WD, Alonso O, Loor JY, et al. Chronic metabolic sequelae of traumatic brain injury: prolonged suppression of somatosensory activation. Am J Physiol Heart Circ Physiol. 2000;279(3):H924–31. doi: 10.1152/ajpheart.2000.279.3.H924 10993751

[69] Ginsberg MD, Zhao W, Alonso OF, Loor-Estades JY, Dietrich WD, Busto R. Uncoupling of local cerebral glucose metabolism and blood flow after acute fluid-percussion injury in rats. Am J Physiol. 1997;272(6 Pt 2):H2859–68. doi: 10.1152/ajpheart.1997.272.6.H2859 9227566

[70] Li FF, Lu LY, Shang SA, Chen HY, Wang P, Haidari NA, et al. Cerebral Blood Flow and Its Connectivity Deficits in Mild Traumatic Brain Injury at the Acute Stage. Neural Plast. 2020;2020. doi: 10.1155/2020/2174371 32684919 PMC7349463

[71] Wang Y, Nelson LD, LaRoche AA, Pfaller AY, Nencka AS, Koch KM, et al. Cerebral Blood Flow Alterations in Acute Sport-Related Concussion. J Neurotrauma. 2016;33(13):1227–36. doi: 10.1089/neu.2015.4072 26414315 PMC4931342

[72] Assaf Y, Beit-Yannai E, Shohami E, Berman E, Cohen Y. Diffusion- and T2-weighted MRI of closed-head injury in rats: a time course study and correlation with histology. Magn Reson Imaging. 1997;15(1):77–85. doi: 10.1016/s0730-725x(96)00246-9 9084028

[73] Ding K, Marquez de la Plata C, Wang JY, Mumphrey M, Moore C, Harper C, et al. Cerebral atrophy after traumatic white matter injury: correlation with acute neuroimaging and outcome. J Neurotrauma. 2008;25(12):1433–40. doi: 10.1089/neu.2008.0683 19072588 PMC2858299

[74] Douglas DB, Iv M, Douglas PK, Anderson A, Vos SB, Bammer R, et al. Diffusion Tensor Imaging of TBI: Potentials and Challenges. Top Magn Reson Imaging. 2015;24(5):241–51. doi: 10.1097/RMR.0000000000000062 26502306 PMC6082670

[75] Hulkower MB, Poliak DB, Rosenbaum SB, Zimmerman ME, Lipton ML. A decade of DTI in traumatic brain injury: 10 years and 100 articles later. AJNR American journal of neuroradiology. 2013;34(11):2064–74. doi: 10.3174/ajnr.A3395 23306011 PMC7964847

[76] Yin B, Li DD, Huang H, Gu CH, Bai GH, Hu LX, et al. Longitudinal Changes in Diffusion Tensor Imaging Following Mild Traumatic Brain Injury and Correlation With Outcome. Front Neural Circuits. 2019;13:28. doi: 10.3389/fncir.2019.00028 31133818 PMC6514143

